# Population-based seroprevalence of HSV-2 and syphilis in Andhra Pradesh state of India

**DOI:** 10.1186/1471-2334-10-59

**Published:** 2010-03-09

**Authors:** John A Schneider, Vemu Lakshmi, Rakhi Dandona, G Anil Kumar, Talasila Sudha, Lalit Dandona

**Affiliations:** 1 Department of Medicine, University of Chicago, Chicago, IL, USA; 2 Department of Health Studies, University of Chicago, Chicago, IL, USA; 3 Department of Microbiology, Nizam's Institute of Medical Sciences, Hyderabad, India; 4 Public Health Foundation of India, New Delhi, India; 5 George Institute for International Health - India, Hyderabad, India; 6 School of Public Health and George Institute for International Health, University of Sydney, Sydney, Australia; 7 Institute for Health Metrics and Evaluation, University of Washington, Seattle, WA, USA

## Abstract

**Background:**

Understanding the prevalence and risk factors for common causes of ulcerative genital disease in the general population would inform current STI syndromic management and HIV testing strategies in high HIV prevalence regions of India.

**Methods:**

Persons 15-49 years old from 32 rural and 34 urban clusters were sampled using a stratified random method to represent adults in the high HIV prevalence Guntur district in Andhra Pradesh state. Interviews were conducted and dry blood spots were collected on 12,617 study participants. Testing for HSV-2 and syphilis was performed.

**Results:**

Adjusted HSV-2 and syphilis seroprevalence rates were 4.70% and 2.08% for men and 7.07% and 1.42% for women. For men, tattooing, >3 lifetime sex partners, tobacco use, and sex with men in the past 6 months were associated with HSV-2 or syphilis (ORs, 1.66-2.95, p < 0.05). Male circumcision was positively associated with HSV-2 infection (OR, 1.37, p = 0.028) though this could be due to residual confounding. In women, greater than one lifetime partner remained significantly associated with HSV-2 in multivariate analysis (OR, 2.61; 95% CI, 1.39-4.87). Among all behavioral risk factors and other covariates in women and men, HIV infection exhibited the strongest association with HSV-2 and syphilis (ORs, 8.2-14.2, p < 0.001). The proportion of individuals with HSV-2 who were HIV infected was less than the proportion with syphilis who were HIV infected (11.8% vs. 22.7%; p = 0.001).

**Conclusions:**

Nearly one in four persons surveyed in this population-based study that were seroprevalent for syphilis, were also HIV infected. Common population risk factors for syphilis, HSV-2 and HIV and high rates of co-seroprevalence suggest that HIV testing, STI testing and service strategies for these would benefit from direct linkage in India.

## Background

Reports from sexually transmitted disease clinics and single health care institutions in India suggest an increase in genital ulcerative sexually transmitted infections and decline in non-ulcerative sexually transmitted infections [1,2]. Amongst sexually transmitted ulcerative infections, *Treponema pallidum*, the bacterium causing syphilis and herpes simplex virus type-2 (HSV-2) have the highest prevalence [1] followed by chancroid and donovanosis which are in decline [3]. HSV-2 seroprevalence rates in the general adult population in India have been reported to range from 7.9 to 18.9% [4-6], translating into 100-200 million individuals who have acquired HSV-2 infection. While HSV-2 can be a cause of significant morbidity through genital ulcer disease, central nervous system disease, and liver involvement, one of the major reasons for interest in HSV-2 is because of its strong association with HIV infection. Despite two recent large randomized trials that demonstrated a lack of an effect of pharmacologic herpes suppression in preventing incident cases of HIV infection [7,8], the continued significant and strong epidemiological evidence linking HIV to HSV-2 continues to support research into novel strategies to mitigate new HIV infection in those who are HSV-2 infected. Moreover, in a study in the state of Karnataka in India, there was significant heterogeneity of HSV-2 infection with higher rates in rural areas and amongst women [6].

The little information available on rates of syphilis in India suggest an incidence rate of 5.4 per 100 person years in a sexually transmitted disease clinic in India from 1993-2000 and prevalence rates as high as 21.9% amongst a convenience sample of long-distance truck-drivers in 2000 [4,5]. Syphilis has also been implicated in increasing susceptibility to HIV infection [9], and HIV prevention interventions based on syphilis and other STI control have, except for in one isolated instance [10], also led to disappointing results [11,12]. Additionally, spikes in syphilis infection has become a concern in men who have sex with men (MSM) communities in other settings [13]. However this has yet to be reported from India, where MSM have one of the highest rates of HIV prevalence amongst groups at high risk of sexual transmission of HIV [14].

Thus HSV-2 and syphilis infection could have a great impact on the Indian population. A better understanding of population level data on the prevalence of HSV-2 and syphilis, and the risk factors for these two important causes of genito-ulcerative diseases could improve public health STI prevention efforts in the country and their integration with HIV prevention efforts.

We report seroprevalence rates and potential risk factors for HSV-2 and syphilis from a population-based study in Guntur district in the south Indian state of Andhra Pradesh. This district and state have one of the highest burdens of HIV infection due to sexual transmission in India [14].

## Methods

### Study population and Setting

Overall 12,617 persons 15-49 years old from 32 rural and 34 urban clusters were sampled using a stratified random method to represent adults in the Guntur district of Andhra Pradesh (AP) India previously described in detail elsewhere [15]. In brief, Guntur district, which had a population of 4.5 million in the 2001 census with 29% urban, was stratified into three regions with different levels of development (literacy, assets, electricity and water assets from census data), and rural and urban areas selected that together would be representative of the district. Within these areas, clusters of 1300-1600 population were selected randomly, and systematic sampling was done to select households in order to get 200-230 eligible persons aged 15-49 years in each cluster. All residents in this age group in each selected household were considered eligible, with a resident defined as someone who had lived in the selected area for the past 6 months or more and a household defined as persons eating from the same kitchen. Assuming a participation rate of 90% of the eligible persons based on pilot studies, we estimated a final sample of about 12 400 persons with approximately equal men-women and rural-urban distribution [15]. Trained field investigators obtained written informed consent from eligible people for participation in the study prior to confidential interview and dry blood spot sampling. As the blood test results would be unlinked with the respondent identity, those interested in knowing their HIV/STI status were referred to the nearest public sector voluntary counselling and testing centre. The protocols and procedures for this study were approved by institutional ethics committees of the Nizam's Institute of Medical Sciences and the Administrative Staff College of India in Hyderabad, India.

### Interviews

Confidential interviews were conducted between September of 2004 and September of 2005 with participants to obtain socio-demographic information and other risk factors that may be associated with the occurrence of sexually transmitted infections [16]. Socio-demographic information included age, sex, education, caste, and a modified standard of living index which is based upon living conditions and ownership of assets [17]. The caste system is a social class system and categories were those used by the Government of India and categorized from highest to lowest: Forward Caste, Backwards Caste, Scheduled Caste and Scheduled Tribe.

Information relevant to this paper included number of lifetime sex partners, frequency of condom use, frequency of alcohol use before sex, blood transfusion history, tattooing, use of recreational drugs, alcohol use, tobacco use, and time spent away from home. For men, female sex worker (FSW) history, having sex with men and self reported circumcision status were obtained.

### Blood samples

A validated finger prick method with a safety lancet was used to take blood samples from each respondent for dry blood spot (DBS) preparation on Whatman No. 3 paper (Whatman International Ltd, Maidstone, UK) [18]. Testing was done using standard methods and kits for detecting HSV-2 IgG (HerpeSelect 2 ELISA IgG, Focus Diagnostics, CA) [19,20] and *Treponema pallidum *IgG (Treponostika sandwich ELISA, bioMérieux, France). The DBS results for HSV-2 and syphilis demonstrated 100% specificity and sensitivity when compared to standard serum testing of 75 samples from STD clinic patients and controls. All positive DBS samples were re-tested for confirmation and a 10% random sample of negative samples was re-tested revealing 100% concordance with the initial result. Both tests indicate lifetime prevalence of disease. Sequential HIV 1/2 antibody, Western blot, p24 antigen and pooled nucleic acid tests from study participants were conducted according to algorithms and methods described elsewhere, [15] with "HIV" in this report referring to HIV-1 and/or HIV-2 seroprevalence and/or acute HIV-1 infection. HIV prevalence and risk factors in this cohort have been described previously [15,21].

### Analysis

The prevalence of HSV-2, syphilis, HSV-2/HIV co-prevalence and syphilis/HIV co-prevalence are reported. We examined the relationship of HSV-2 and syphilis seroprevalence with sociodemographic and risk variables (hygiene, substance use, sexual risk, HIV, and STI) in bivariate analyses for men and women separately. We then utilized a stepwise approach by creating two separate initial logistic regression models for HSV-2 and syphilis which included either sociodemographic or risk variables significant at the p < 0.05 level from bivariate analyses. Variables from the sociodemographic and risk models that remained statistically significant in the initial regression analyses were then combined into one final multiple logistic regression model for HSV-2 and syphilis separately for men and women. HIV serostatus was also examined as an outcome variable in separate models including HSV-2, syphilis and significant risk and sociodemographic variables. Data were analyzed using STATA software version 9.0 (StataSoft Corp, Austin TX, USA).

## Results

Of the final 13,838 15-49-year-olds sampled, 12,617 (91.2%) gave a blood sample and interview, 6% did not participate, and 2.8% provided an interview only. Of this final analytic sample 12,617 study participants who provided a blood sample, 6317 (50.1%) were rural residents, 6382 (50.6%) were women, 4582 (36.3%) <25 years of age, and 4310 (34.2%) had not had any schooling.

### Seroprevalence

Population-based proportions of HSV-2 and syphilis for the two sexes and urban and rural residence are presented in Table 1. The overall seroprevalence of HSV-2 and syphilis adjusted for the age, sex and rural-urban distribution of Guntur district population was 5.87% (95% CI, 4.28-7.46) and 1.75% (95% CI, 1.33-2.17), respectively. HSV-2 and syphilis seroprevalence increased with age, decreased with increasing education and improved SLI, and was higher in urban residents. These relationships were maintained when stratified by gender. However, women were more likely to have HSV-2 (OR, 1.54; 95% CI, 1.34 - 1.78) and less likely to have syphilis (OR, 0.56; 95% CI, 0.42 - 0.74) when compared to men adjusting for sociodemographic characteristics that were included in the final model.

**Table 1:** Population-based rates of HSV-2 and syphilis seroprevalence in Guntur District, Andhra Pradesh, South India (N = 12,617).*

HSV-2										
	**Men**				**Women**				**Total**	

	**Participants**	**HSV Positive (%)**	**Adjusted HSV prevalence %**	**95% confidence interval; design effect**	**Participants**	**HSV Positive (%)**	**Adjusted HSV prevalence %**	**95% confidence interval; design effect**	**Adjusted HSV rate %**	**95% confidence interval; design effect**

Rural	3139	120(3.82)	3.20	1.80-4.60; 4.30	3178	167(5.25)	4.56	2.55-6.57; 6.56	3.87	2.24-5.50; 9.88

Urban	3096	243(7.85)	8.15	6.03-10.27; 5.14	3204	417(13.01)	12.77	9.78-15.76; 6.67	10.44	8.05-12.83; 10.08

**Total**	6235	363(5.82)	4.70	3.36-6.04; 5.40	6382	584(9.15)	7.07	5.06-9.08; 8.11	**5.87**	4.28-7.46; 12.08

**Syphilis**										

	**Men**				**Women**				**Total**	

	**Participants**	**Syphilis Positive (%)**	**Adjusted syphilis prevalence %**	**95% confidence interval; design effect**	**Participants**	**Syphilis Positive (%)**	**Adjusted syphilis prevalence %**	**95% confidence interval; design effect**	**Adjusted syphilis rate %**	**95% confidence interval; design effect**

Rural	3139	55(1.75)	1.71	0.90-2.52; 3.01	3178	41(1.29)	1.38	0.90-1.86; 1.30	1.55	0.97-2.13; 3.38

Urban	3096	79(2.55)	2.93	2.05-3.81; 2.54	3204	45(1.40)	1.51	1.03-1.99; 1.57	2.22	1.61-2.83; 3.34

**Total**	6235	134(2.15)	2.08	1.48-2.68; 2.75	6382	86(1.35)	1.42	1.08-1.76; 1.41	**1.75**	1.33-2.17; 3.32

*Overall HSV-2 and syphilis seroprevalence adjusted for age, sex, gender and rural/urban distribution of the population in Guntur district.

### Risk Factors

In bivariate analyses, risk variables associated with HSV-2 or syphilis in men included lack of toilet facility at home, history of transfusion, tattooing, circumcision, tobacco use, alcohol consumption, multiple lifetime female partners, ever having had sex with female sex workers, condom use, ever having had sex with men, using alcohol before sex, HSV-2 or syphilis and HIV 1/2 seroprevalence. In women, history of having a toilet facility at home, history of transfusion, any recreational drug use, ever chewed tobacco, condom use, having had more than one lifetime partner, HSV-2 or syphilis and HIV status were associated with HSV-2 or syphilis seroprevalence. Variables that remained statistically significant after separate initial multiple logistic regression models for behavioral and sociodemographic variables with outcome of HSV-2 and syphilis are combined into final models for men (Table 2) and women (Table 3) separately. The range of R^2 ^for final models was 0.14-0.18.

**Table 2:** Combined multiple logistic regression model including socio-demographic and behavioral variables for association with seroprevalent HSV-2 and syphilis in men.

Factor	N (% of Total)	HSV-2*			Syphilis †		
		**N (% HSV-2)**	**Odds of having HSV-2 with univariate analysis (95% CI)**	**Adjusted odds of having HSV-2 with multiple logistic regression (95% CI)**	**N (% Syphilis)**	**Odds of having syphilis with univariate analysis (95% CI)**	**Adjusted odds of having syphilis with multiple logistic regression (95% CI)**

**Men (N = 6,235) **‡							

*Sociodemographics*							

Age (years)							

15-19	1175 (18.8)	30 (2.6)	1.00 (ref)	1.00 (ref)	6 (0.5)	1.00 (ref)	

20-24	1086 (17.4)	42 (3.9)	1.54 (0.95-2.47)	1.07 (0.62-1.84)	14 (1.3)	2.54 (0.97-6.65)	

25-29	972 (15.6)	56 (5.8)	2.33 (1.49-3.67)	1.36 (0.77-2.40)	28 (2.9)	5.78 (2.38-14.01)	

30-34	833 (13.4)	53 (6.4)	2.59 (1.64-4.10)	1.13 (0.62-2.06)	24 (2.9)	5.78 (2.35-14.20)	

35-39	842 (13.5)	70 (8.3)	3.46 (2.23-5.36)	1.74 (0.97-3.11)	25 (3.0)	5.96 (2.43-14.60)	

40-44	628 (10.1)	43 (6.8)	2.81 (1.74-4.52)	1.56 (0.84-2.88)	19 (3.0	6.08 (2.42-15.30)	

45-49	699 (11.2)	69 (9.9)	4.18 (2.69-6.49)	2.60 (1.46-4.62) §	18 (2.6)	5.15 (2.03-13.04)	

Standard of Living Index ¶							

0-16	1564 (25.1)	107 (6.8)	1.00 (ref)	1.00 (ref)	51 (3.3)	1.00 (ref)	

17-22	1593 (25.5)	100 (6.3)	0.91 (0.69-1.21)	0.95 (0.69-1.29)	34 (2.1)	0.65 (0.42-1.0)	

23-29	1529 (24.5)	89 (5.8)	0.84 (0.63-1.13)	0.95 (0.68-1.33)	23 (1.5)	0.45 (0.28-0.75)	

30-54	1548 (24.8)	67 (4.3)	0.62 (0.45-0.84)	0.64 (0.43-0.95) §	26 (1.7)	0.51 (0.31-0.82)	

Education							

None	1573 (25.2)	103 (6.5)	1.00 (ref)	1.00 (ref)	47 (3.0)	1.00 (ref)	

Class 1-10	3180 (51.0)	203 (6.4)	0.97 (0.76-1.24)	1.12 (0.85-1.49)	73 (2.3)	0.76 (0.53-1.11)	

Class 11-12	518 (8.3)	24 (4.6)	0.69 (0.44-1.09)	1.18 (0.71-1.97)	3 (0.6)	0.19 (0.06-0.61)	

More than Class 12	960 (15.4)	33 (3.4)	0.51 (0.34-0.76)	0.82 (0.50-1.33)	11 (1.1)	0.38 (0.19-0.73)	

Occupation							

Other than categories below	4897 (78.5)	253 (5.2)	1.00 (ref)		86 (1.8)		1.00 (ref)

Transport Related	438 (7.0)	39 (8.9)	1.79 (1.26-2.55)		24 (5.5)	3.24 (2.04-5.16)	2.18 (1.30-3.65)

Field Related	822 (13.2)	62 (7.5)	1.50 (1.12-2.0)		23 (2.8)	1.61 (1.01-2.57)	1.32 (0.81-2.17)

Begging/Sex Work	18 (0.3)	5 (27.8)	7.06 (2.50-19.96)		0 (0)	Model does not converge	Model does not converge

Unemployed	60 (1.0)	4 (6.7)	1.31 (0.47-3.64)		1 (1.7)	0.95 (0.13-6.9)	0.74 (0.09-5.77)

Caste							

Forward	2,323 (37.3)	115 (5.0)	1.00 (ref)		36 (1.5)	1.00 (ref)	1.00 (ref)

Backward	1,532 (24.6)	98 (6.4)	1.31 (0.99-1.73)		47 (3.1)	2.01 (1.30-3.12)	1.66 (1.02-2.71) §

Scheduled	1285 (20.6)	80 (6.2)	1.27 (0.95-1.71)		27 (2.1)	1.36 (0.82-2.26)	1.01 (0.57-1.79)

Scheduled Tribe	219 (3.5)	8 (3.7)	0.73 (0.35-1.51)		3 (1.4)	0.88 (0.27-2.89)	0.61 (0.18-2.13)

None	876 (14.0)	62 (7.1)	1.46 (1.06-2.01)		21 (2.4)	1.56 (0.91-2.69)	1.43 (0.79-2.59)

Place of Residence							

Rural	3139 (50.3)	120 (3.8)	1.00 (ref)	1.00 (ref)	55 (1.8)	1.00 (ref)	

Urban	3,096 (49.7)	243 (7.8)	2.14 (1.71-2.68)	2.21 (1.71-2.86) ||	79 (2.6)	1.47 (1.04-2.08)	

*Health and Hygiene*							

History of Tattoo							

No	5747 (92.2)	301 (5.2)	1.00 (ref)	1.00 (ref)	110 (1.9)	1.00 (ref)	

Yes	483 (7.8)	62 (12.8)	2.66 (1.99-3.56)	1.66 (1.18-2.33) §	24 (5.0)	2.68 (1.71-4.21)	

History of Transfusion							

No	6073 (97.5)	343 (5.6)	1.00 (ref)		130 (2.1)	1.00 (ref)	

Yes	156 (2.5)	20 (12.8)	1.58 (1.20-2.07)		4 (2.6)	1.20 (0.44-3.30)	

Circumcision							

Uncircumcised	5156 (83.6)	278 (5.4)	1.00 (ref)	1.00 (ref)	107 (2.1)		

Circumcised	1011 (16.4)	80 (7.9)	1.51 (1.16-1.95)	1.37 (1.03-1.82) §	25 (2.5)	1.20 (0.77-1.86)	

Toilet Facility at Home							

Own Flush	1547 (24.8)	83 (5.4)	1.00 (ref)		21 (1.4)	1.00 (ref)	

Share Flush/Own Pit Toilet	1848 (29.6)	129 (7.0)	1.26 (1.01-1.57)		42 (2.3)	2.82 (1.34-5.90)	

Share Pit Toilet	307 (4.9)	17 (5.5)	0.90 (0.60-1.34)		11 (3.6)	1.85 (0.57-6.05)	

None	2491 (40.0)	126 (5.1)	0.66 (0.52-0.83)		60 (2.4)	2.62 (1.27-5.43)	

Chew or Smoke Tobacco							

Never	2722 (43.7)	111 (4.1)	1.00 (ref)		28 (1.0)	1.00 (ref)	1.00 (ref)

Previous	369 (5.9)	33 (8.9)	2.31 (1.54-3.46)		9 (2.4)	2.41 (1.13-5.14)	1.47 (0.65-3.34)

Current	3137 (50.3)	219 (7.0)	1.77 (1.40-2.23)		97 (3.1)	3.07 (2.01-4.69)	2.07 (1.26-3.41) §

*Sexual Risk*							

Lifetime Female Sex Partners							

0	1386 (22.5)	35 (2.5)	1.00 (ref)	1.00 (ref)	5 (0.4)	1.00 (ref)	1.00 (ref)

1	1671 (27.1)	73 (4.4)	1.62 (1.10-2.40)	1.23 (0.72-2.09)	33 (2.0)	4.19 (1.85-9.50)	2.96 (1.00-8.81)

2-3	1549 (25.1)	92 (5.9)	2.24 (1.54-3.28)	1.52 (0.89-2.61)	36 (2.3)	4.95 (2.19-11.15)	2.93 (0.97-8.83)

4-7	881 (14.1)	71 (8.8)	3.44 (2.31-5.12)	2.14 (1.22-3.76) §	32 (4.0)	8.60 (3.78-19.59)	4.50 (1.47-13.81) §

>7	672 (10.9)	87 (11.6)	4.68 (3.18-6.88)	2.44 (1.39-4.29) §	26 (3.5)	7.49 (3.23-17.33)	2.74 (0.86-8.73)

Sex with Men							

Never	6029 (97.9)	338 (5.6)	1.00 (ref)	1.00 (ref)	127 (2.1)	1.00 (ref)	

> 6 months ago	85 (1.4)	9 (10.6)	1.99 (0.99-4.01)	1.27 (0.57-2.82)	2 (2.4)	1.12 (0.27-4.60)	

Within last 6 months	47 (0.8)	11 (23.4)	5.14 (2.60-10.20)	2.95 (1.27-6.82) §	3 (6.4)	3.17 (0.97-10.34)	

*HIV/STI Serostatus*							

HIV 1/2	130 (2.1)	66 (51.6)	20.17 (14.03-28.99)	14.16 (9.37-21.40) ||	29 (22.3)	16.41 (10.40-25.88)	8.70 (4.99-15.18) ||

HSV-2	363 (5.8)	-			33 (9.1)	5.71 (3.80-8.60)	2.35 (1.42-3.90) ||

Syphilis	134 (2.2)	33 (24.6)	5.71 (3.80-8.60)	2.08 (1.25-3.46) §	-		

*Variables included in final HSV-2 model: tatoo, circumcision, lifetime female sex partners, sex with men, HIV 1/2 serostatus, syphilis serostatus, age, SLI, education, place of residence.

†Variables included in final syphilis model: chew or smoke tobacco, lifetime female sex partners, HIV 1/2 serostatus, HSV-2 serostatus, occupation, caste.

‡Data missing: 5 for tatoo, 6 for transfusion, 68 for circumcision, 7 for tobacco, 76 for female sex partners, 74 for sex with men, 1 for SLI, 4 for education and 265 for time away from home

Reflects number who responded to all items in table; the total for sub-categories may not add up to 12,617 due to some missing data.

§ p < 0.05 in final model

|| p < 0.001 in final model

¶Based on living conditions and assets, which was adapted from an index used previously by the National Health and Family Life Survey in India.[14]

**Table 3:** Combined multiple logistic regression model including socio-demographic and behavioral variables for association with seroprevalent HSV-2 and syphilis in women.

Factor	N (% of Total)	HSV-2*			Syphilis †		
		**N (% HSV-2)**	**Odds of having HSV-2 with univariate analysis (95% CI)**	**Adjusted odds of having HSV-2 with multiple logistic regression (95% CI)**	**N (% Syphilis)**	**Odds of having syphilis with univariate analysis (95% CI)**	**Adjusted odds of having syphilis with multiple logistic regression (95% CI)**

**Women (N = 6,382) **‡							

*Sociodemographics*							

Age (years)							

15-19	1,159 (18.2)	48 (4.1)	1.00 (ref)	1.00 (ref)	6 (0.5)	1.00 (ref)	1.00 (ref)

20-24	1,162 (18.2)	62 (5.3)	1.30 (0.89-1.92)	0.91 (0.58-1.44)	14 (1.2)	2.34 (0.90-6.12)	1.55 (0.54-4.45)

25-29	1,027 (16.1)	88 (8.6)	2.17 (1.51-3.12)	1.67 (1.07-2.61) §	9 (0.9)	1.70 (0.60-4.79)	0.85 (0.27-2.68)

30-34	886 (13.9)	110 (12.4)	3.28(2.31-4.66)	2.51 (1.61-3.90) ||	19 (2.1)	4.21 (1.67-10.59)	1.88 (0.65-5.44)

35-39	864 (13.5)	118 (13.7)	3.66 (2.58-5.19)	3.13 (2.01-4.86) ||	13 (1.5)	2.94 (1.11-7.75)	1.45 (0.49-4.33)

40-44	757 (11.9)	99 (13.1)	3.48 (2.43-4.98)	2.77 (1.76-4.39) ||	19 (2.5)	4.95 (1.97-12.45)	2.90 (1.01-8.36) §

45-49	527 (8.3)	59 (11.2)	2.92 (1.96-4.34)	2.12 (1.29-3.51) §	6 (1.1)	2.21 (0.71-6.89)	1.39 (0.40-4.88)

Standard of Living Index§							

0-16	1,705 (26.7)	172 (10.1)	1.00 (ref)	1.00 (ref)	33 (1.9)	1.00 (ref)	

17-22	1,603 (25.1)	154 (9.6)	0.95 (0.75-1.19)	0.87 (0.66-1.15)	25 (1.6)	0.80 (0.48-1.36)	

23-29	1,522 (23.8)	144 (9.5)	0.93 (0.74-1.18)	0.93 (0.69-1.26)	16 (1.1)	0.54 (0.30-0.98)	

30-54	1,552 (24.3)	114 (7.3)	0.71 (0.55-0.90)	0.61 (0.42-0.90) §	12 (0.8)	0.39 (0.20-0.77)	

Education							

None	2737 (42.9)	296 (10.8)	1.00 (ref)		48 (1.8)	1.00 (ref)	

Class 1-10	2825 (44.3)	241 (8.5)	0.77 (0.64-0.92)		35 (1.2)	0.70 (0.45-1.09)	

Class 11-12	335 (5.2)	20 (6.0)	0.52 (0.33-0.84)		3 (0.9)	0.51 (0.16-1.63)	

More than Class 12	484 (7.6)	27 (5.6)	0.52 (0.33-0.84)		0 (0.0)	Model doesn't converge	

Occupation							

Other than those below	5666 (88.8)	459 (8.1)	1.00 (ref)		73 (1.3)	1.00 (ref)	

Field Related	92 (1.4)	15 (6.3)	2.21 (1.26-3.87)		4 (4.4)	3.53 (1.26-9.88)	

Begging/Sex Work	19 (0.3)	5 (26.3)	4.05 (1.45-11.30)		1 (5.3)	4.32 (0.57-32.8)	

Unskilled Labor	605 (9.5)	105 (17.4)	2.38 (1.89-3.0)		8 (1.4)	1.17 (0.58-2.36)	

Caste							

Forward	2398 (37.6)	198 (8.3)	1.00 (ref)	1.00 (ref)	25 (1.0)	1.00 (ref)	

Backward	1572 (24.6)	164 (10.4)	1.29 (1.04-1.61)	1.03 (0.80-1.33)	31 (2.0)	1.91 (1.12-3.25)	

Scheduled	1315 (20.6)	125 (9.5)	1.17 (0.92-1.48)	1.29 (0.98-1.71)	18 (1.4)	1.32 (0.72-2.42)	

Scheduled Tribe	220 (3.4)	22 (10.0)	1.23 (0.78-1.96)	1.03 (0.61-1.73)	1 (0.5)	0.43 (0.06-3.21)	

None	877 (13.7)	75 (8.6)	1.04 (0.79-1.37)	0.70 (0.51-0.96) §	11 (1.3)	1.21 (0.59-2.46)	

Place of Residence							

Rural	3,178 (49.8)	167 (5.3)	1.00 (ref)	1.00 (ref)	41 (1.3)	1.00 (ref)	

Urban	3,204 (50.2)	417 (13.0)	2.70 (2.24-3.25)	3.62 (2.76-4.76) ||	45 (1.4)	1.09 (0.71-1.67)	

*Health and Hygiene*							

History of Transfusion							

No	5,873 (92.0)	517 (8.8)	1.00 (ref)		81 (1.4)	1.00 (ref)	

Yes	507 (7.9)	67 (13.2)	1.28 (0.95-1.72)		5 (1.0)	0.71 (0.29-1.77)	

Toilet Facility at Home							

Own Flush	1,506 (23.7)	146 (9.7)	1.00 (ref)		9 (0.6)	1.00 (ref)	

Share Flush/Own Pit Toilet	1,982 (31.1)	236 (11.9)	1.26 (1.01-1.57)		33 (1.7)	0.66 (0.52-0.83)	

Share Pit Toilet	363 (5.7)	32 (8.8)	0.90 (0.60-1.34)		4 (1.1)	1.85 (0.57-6.05)	

None	2,512 (39.5)	166 (6.6)	0.66 (0.52-0.83)		39 (1.6)	2.62 (1.27-5.43)	

Chew Tobacco							

Never	6,305 (98.9)	566 (9.0)	1.00 (ref)		83 (1.3)	1.00 (ref)	

Ever	67 (1.1)	17 (25.4)	1.77 (0.94-3.33)		2 (3.0)	2.31 (0.56-9.58)	

*Sexual Risk*							

Lifetime Male Partners							

0	759 (12.2)	30 (4.0)	1.00 (ref)	1.00 (ref)	2 (0.3)	1.00 (ref)	

1	4,909 (78.9)	452 (9.2)	2.47 (1.69-3.60)	1.57 (0.91-2.73)	62 (1.3)	4.82 (1.18-19.76)	

>1	551 (8.9)	88 (15.6)	4.68 (3.04-7.20)	2.61 (1.39-4.87) §	18 (3.3)	13.23 (3.06-57.26)	

Condom Use							

No sex in last 6 months	1449 (24.8)	143 (9.9)	1.00 (ref)	1.00 (ref)	17 (1.2)	1.00 (ref)	

Sometimes/Often Use Condoms	124 (1.0)	21 (16.9)	1.86 (1.13-3.07)	1.12 (0.60-2.11)	4 (3.2)	2.81 (0.93-8.48)	

Never Use Condoms	4,653 (74.7)	406 (8.7)	0.87 (0.71-1.07)	0.69 (0.53-0.91) §	61 (1.3)	1.12 (0.65-1.92)	

*HIV/STI serostatus*							

HIV 1/2	111 (1.7)	66 (59.5)	16.29 (11.03-24.04)	14.07 (8.84-22.40) ||	21 (18.9)	22.28 (13.06-38.01)	8.19 (4.29-15.65) ||

HSV-2	584 (9.2)	-			44 (7.5)	11.17 (7.25-17.20)	6.33 (3.79-10.58) ||

Syphilis	86 (1.3)	44 (51.2)	11.17 (7.25-17.20)	6.66 (3.88-11.42) ||	-		

*Variables included in final HSV-2 model: lifetime male partners, condom use, HIV 1/2 serostatus, syphilis serostatus, age, SLI, caste, place of residence.

†Variables included in final syphilis model: HIV 1/2 serostatus, HSV-2 serostatus, age

‡ Data missing for women: 2 for transfusion, 19 for toilet facility, 10 for chew tobacco, 163 for lifetime male partners, 156 for condom use, and 1 for education Reflects number who responded to all items in table; the total for sub-categories may not add up to 12,617 due to some missing data.

§p < 0.05 in final model

|| p < 0.001 in final model

Based on living conditions and assets, which was adapted from an index used previously by the National Health and Family Life Survey in India.[14]

In these final models, of all substance use related items (tobacco use, drinking alcohol, use of alcohol before sex, and use of other recreational drugs) for men, none demonstrated a significant association with HSV-2 (ORs, 0.62-1.64; p values, 0.053-0.52) and only current tobacco use was marginally associated with syphilis seroprevalence (OR, 2.07; 95% CI, 1.26-3.41). Men who never used condoms had similar rates of HSV-2 and syphilis to men who had never had sex or last sex was more than 6 months prior to interview (ORs, 0.39-1.02; p values, 0.059-0.96).

Variables that were associated with HSV-2 in women and remained significant after multivariate modeling included greater than one lifetime partner (OR, 2.61; 95% CI, 1.39-4.87), HIV (OR, 14.07; 95% CI, 8.84-22.40) and syphilis (OR, 6.66; 95% CI, 3.88-11.42). Never using a condom was negatively associated with HSV-2 amongst women who had ever had sex (OR, 0.69; 95% CI, 0.53-0.91).

The relationship between circumcision and HSV-2 in men was a bit more complicated as HSV-2 seroprevalence rates were higher in circumcised men in bivariate analyses of all men (7.9% vs 5.3%; p = 0.002), sexually active men (8.7% vs. 6.4%; p = 0.02) and sexually inactive men (5.6% vs. 1.9%; p = 0.001). In multivariate analysis, male circumcision demonstrated a significant association with HSV-2 (OR, 1.37; 95% CI, 1.03-1.82) but not with syphilis (OR, 1.20; 95% CI, 0.77-1.66). Circumcised men were likely to have more lifetime sexual partners, including FSWs, than those who were not circumcised (8.6 partners vs. 5.0 partners; p = 0.007) and were more likely to have had contact with a FSW (35.5% vs. 22.7%; p < 0.001). When adding risk factors significant in bivariate analyses sequentially to our final HSV-2 model in Table 2, ever having contact with a FSW decreased the point estimate of circumcision by the greatest magnitude. Of the men in this study, 899 (14.4%) were Muslim of whom 96.4% reported being circumcised, whereas only 2.9% of the non-Muslim men reported being circumcised. Religion was not used in the multivariate model as it was highly associated with male circumcision. The latter was included in the model as it was expected to have a more direct relation with sexually transmitted infections and it may be a risk factor that is modifiable in this setting.

### Relationship of HIV seroprevalence with HSV-2 and syphilis

Co-seroprevalence rates of HSV-2 or syphilis with HIV infection and proportions of HSV-2 or syphilis infected with HIV are presented in Table 4. The proportion of HSV-2 individuals who were HIV infected was less when compared to the proportion of syphilis individuals who were HIV infected (11.8% vs. 22.7%; Chi-square 10.48; p = 0.001). HIV 1/2 seroprevalence had a strong association with HSV-2 and syphilis in men (OR, 13.98; 95% CI 9.27-21.10) and (OR, 8.58; 95% CI 4.93-14.92), respectively.

**Table 4:** Population-based gender rates of HSV-2/HIV and syphilis/HIV co-seroprevalence in Guntur District, Andhra Pradesh, South India.*

HSV-2										
	**Men**				**Women**				**Total**	

	**HSV positive**	**HSV-2/HIV co-prevalence**	**Proportion of HSV-2 co-prevalence %**	**Adjusted HSV-2/HIV co-prevalence**	**HSV-2 positive**	**HSV-HIV co-prevalence**	**Proportion of HSV-2/HIV** **%**	**Adjusted HSV-2/HIV co-prevalence**	**Proportion of HSV-2 co-prevalence %**	**Adjusted co-prevalence %**

**Total**	363	66	18.2	0.85	584	66	11.3	0.84	**11.8**	**0.85**

**Syphilis**										

	**Men**				**Women**				**Total**	

	**Syphilis positive**	**Syphilis/HIV co-prevalence**	**Proportion of Syphilis co-prevalence %**	**Adjusted syphilis/HIV co-prevalence**	**Syphilis positive**	**Syphilis-HIV co-prevalence**	**Proportion of Syphilis/HIV %**	**Adjusted syphilis/HIV co-prevalence**	**Proportion of Syphilis co-prevalence %**	**Adjusted co-prevalence %**

**Total**	134	29	21.6	0.35	86	21	24.4	0.29	**22.7**	**0.32**

*Overall HSV-2/HIV and syphilis/HIV seroprevalence adjusted for age, sex, gender and rural/urban distribution of the population in Guntur district

## Discussion

This report examines HSV-2 and syphilis seroprevalence and associated risk factors in a large population-based sample, with a high participation rate, in a high HIV prevalent district [22] within a high prevalence state [14] of India. Seroprevalence of HSV-2 and syphilis demonstrated rural urban and gender differences. Seroprevalence for both HSV-2 and syphilis was higher in urban areas, HSV-2 was higher amongst women and syphilis was slightly higher in men.

Though less information from population based studies exists on rates of syphilis infection, estimates suggest that South Asia has the highest number of cases in the world [23]. Moreover, the burden of HSV-2 and syphilis infection extrapolated from these rates is high and supports current national efforts to address STI disease in the country. In general terms while the overall prevalence of HSV-2 is significantly lower than many other countries in sub-Saharan Africa, the ratio of HSV-2/HIV seroprevalence at 2-3 is roughly similar between our study and HSV-2/HIV seroprevalence ratios found in other populations in Africa [24].

Marked heterogeneity in syphilis and HSV-2 at the district and state level can become apparent when comparing burden of STIs across studies despite treatment policies that often remain the same across geographic regions [25]. For example, syphilis seroprevalence in our study was about four times that found in another study in a district in the neighboring state Karnataka while HSV-2 in our district level population was nearly 1/3 of that found in the district in Karnataka [6]. HSV-2 and syphilis seroprevalence rates were higher in urban areas and rates of syphilis were higher in men than in women in our study, while opposite trends were found in the Karnataka study. However, testing algorithms and testing kits differed between the two studies as well. While some of the differences in seroprevalence rates may be methodological, the heterogeneous nature of the STI epidemic in India, could explain the differences in findings at the district level, warranting programmatic prevention and treatment programs to reflect local conditions.

Our finding of a positive relationship between circumcised status and HSV-2 seroprevalence differs from the inverse relationship between circumcision and HIV found in Indian populations described previously [26], and that of a randomized controlled trial [27]. Moreover, uncircumcised status demonstrated a strong positive association with HIV seroprevalence within this same cohort in data described elsewhere [21]. A positive association between HSV-2 and circumcision has also been described in other epidemiologic work [28], and a lack of association has also been reported [29]. Results from a randomized controlled trial in Uganda, demonstrate a protective effect of circumcision on HSV-2 [30], however, potentially less protection than has been previously been found for HIV [27]. The role of sexual partnering patterns at the population level may play a significant role for HSV-2 acquisition, with cumulative cases as an important HSV-2 risk factor. Observational studies examining circumcision and STIs in India should take into consideration potential sexual partnering differences between circumcised and uncircumcised members of the population, and residual confounding, which may have resulted in the positive association of male circumcision with HSV-2 seroprevalence in our cross-sectional study.

We found lower odds of HSV-2 in women who reported never using condom. The explanation could be a higher frequency of condom use currently by those who are at high risk of HSV-2 (risk compensation) and/or a bias towards higher reporting of condom use by those who are at higher risk of HIV/STI. We have previously reported a similar association between never using condom and low HIV among both men and women in this study [21].

This population level STI co-seroprevalence with HIV data highlights and builds upon existing data from STI clinics of high HIV/STI co-seroprevalence in India [31,32]. Our finding that 23% of those seroprevalent for syphilis and 12% seroprevalent for HSV-2, were also found to be HIV infected has important implications for STI syndromic management and HIV testing strategies practiced in India. Because syndromic management is not based on a proven diagnosis, such population based data is needed to inform local treatment algorithms. For example in this district in Andhra Pradesh with potentially less HSV-2, syndromic management that utilizes less acyclovir therapy may be of higher priority than in a neighboring state or district. Refinement of syndromic management algorithms might include studies that examine the etiology of genital ulceration and rates of asymptomatic infection or viral shedding.

Furthermore, recent national guidelines for strengthening STI/RTI services within government institutions [33] have lacked any mention of HIV testing at these centers. In one recent study from an STI clinic in Pune India, the majority of cases where both syphilis and HIV were acquired were detected at the same follow up visit suggesting co-transmission of HIV and syphilis [5]. Current guidelines for the operational management of RTI/STI clinics suggest that patients seeking STI care and found to have an STI or with symptoms are to be referred to local Integrated Counseling and Testing Centers (ICTC) for HIV testing. However, evaluation of the potential effectiveness of this approach has not been done yet. While the numbers of ICTCs in Andhra Pradesh state has increased dramatically to 661 centers [34], quality and accessibility has not been documented publicly and barriers to seeking voluntary counseling and testing have been reported [35]. Additions to government STI syndromic management algorithms could include a longer duration and increased doses of penicillin in HIV infected persons, for example. Additionally, picking up acute or early HIV infection during this time is critical to following through with partner notification procedures. Improvements in sensitivity, specificity and a decrease in the window period with newer rapid third generation whole blood kits now commercially available in parts of the country, suggest that HIV testing during presentation with STI symptoms could be an ideal time to pick up new cases of HIV and prevent loss to follow-up of patients referred out for HIV testing.

There were several limitations of this study. The sampling frame was limited to one district within Andhra Pradesh and we are unable to extrapolate these findings to other geographic regions. However, this information is useful as it comes from a high HIV prevalent district within a high HIV prevalent state and demonstrates relatively low levels of HSV-2 and syphilis. Findings from local data can impact our understanding of disease at the national level as evidenced from the previously described HIV prevalence from this study [15] which played a major role in the downward estimation of HIV burden for the country [36]. Additionally, without further clinical history and non-specific syphilis testing, we were unable to determine whether results from syphilis IgG testing were consistent with active, incubating, treated or convalescent infection. However, the yield of active syphilis would be less helpful from a population-based study as active cases of syphilis would be even less frequent in such a setting. Also high titers would have been difficult to interpret without a clinical history given that untreated secondary or tertiary syphilis could be present, however, both conditions would be insignificant in relation to transmission dynamics as syphilis can be non-communicable after 1-4 years [16].

Herpes and syphilis suppression and control as HIV prevention interventions have not been found to be effective [7,8,37]. However, additional examination of this HIV prevention strategy continues given the strong relationship between these sexually transmitted infections and HIV. However, there may be specific situations where such control or suppression strategies are used, such as during pregnancy, to prevent the devastating potential sequelae of neonatal herpes infection or intrapartum syphilis infection. HSV-2 control may be less important in this setting as an HIV prevention strategy as the HIV epidemic has not spread widely and levels of HSV-2 infection in India are relatively low.

## Conclusion

This study provides data on the burden of HSV-2 and syphilis at the population level and its associations in a high HIV prevalence part of India. The strong association between HSV-2, syphilis and HIV seroprevalence in this population-based study suggests that acceleration of direct linkages between STI testing and HIV counseling and testing would be useful in enhancing the control of STIs and HIV in India.

## Competing interests

The authors declare that they have no competing interests.

## Authors' contributions

The parent study from which this report is prepared was conceived by LD with major contributions to the design by VL and RD. JAS performed the statistical analysis for this report and drafted the manuscript in collaboration with RD and LD. GAK contributed to the statistical analysis. TS contributed to the laboratory analysis. All authors approved the final version of the manuscript.

## Pre-publication history

The pre-publication history for this paper can be accessed here:

http://www.biomedcentral.com/1471-2334/10/59/prepub
